# The prosocial personality and its facets: genetic and environmental architecture of mother-reported behavior of 7-year-old twins

**DOI:** 10.3389/fpsyg.2015.00112

**Published:** 2015-02-13

**Authors:** Ariel Knafo-Noam, Florina Uzefovsky, Salomon Israel, Maayan Davidov, Caroyln Zahn-Waxler

**Affiliations:** ^1^Department of Psychology, The Hebrew University of Jerusalem, Jerusalem, Israel; ^2^Department of Psychiatry, University of Cambridge, Cambridge, UK; ^3^School of Social Work and Social Welfare, The Hebrew University of Jerusalem, Jerusalem, Israel; ^4^University of Wisconsin at Madison, Madison, WI, USA

**Keywords:** prosocial behavior, empathy, developmental psychology, behavior genetics, personality

## Abstract

Children vary markedly in their tendency to behave prosocially, and recent research has implicated both genetic and environmental factors in this variability. Yet, little is known about the extent to which different aspects of prosociality constitute a single dimension (the prosocial personality), and to the extent they are intercorrelated, whether these aspects share their genetic and environmental origins. As part of the Longitudinal Israeli Study of Twins (LIST), mothers of 183 monozygotic (MZ) and dizygotic (DZ) 7-year-old twin pairs (51.6% male) reported regarding their children’s prosociality using questionnaires. Five prosociality facets (sharing, social concern, kindness, helping, and empathic concern) were identified. All five facets intercorrelated positively (*r* > 0.39) suggesting a single-factor structure to the data, consistent with the theoretical idea of a single prosociality trait. Higher MZ than DZ twin correlations indicated genetic contributions to each prosociality facet. A common-factor-common-pathway multivariate model estimated high (69%) heritability for the common prosociality factor, with the non-shared environment and error accounting for the remaining variance. For each facet, unique genetic and environmental contributions were identified as well. The results point to the presence of a broad prosociality phenotype, largely affected by genetics; whereas additional genetic and environmental factors contribute to different aspects of prosociality, such as helping and sharing.

## INTRODUCTION

Humans show a rich and complex array of positive behaviors, attitudes, and emotions directed towards others, referred to here as *prosociality*. This prosocial tendency has been described as the result of an evolutionary drive towards cooperation between group members and kin (Nowak, 2006; de Waal, 2008).

Prosociality is a complex, multidimensional construct (Padilla-Walker and Carlo, 2014). At the affective level, the prosocial repertoire of humans includes *empathy*, an other-oriented affective tendency to comprehend and share the emotional states of others (Eisenberg et al., 2006; see also their discussion of the related constructs of *sympathy* and *compassion*). At the behavioral level, *prosocial behavior* is defined as a voluntary behavior enacted with the intent of benefiting others (Eisenberg et al., 2006). There are many kinds of prosocial behaviors, with most concerning either *sharing* (giving from personal resources), providing instrumental *help*, or *comforting* (supporting others emotionally in times of distress). Finally, at the attitudinal level, prosociality includes prosocial values such as benevolence (Schwartz, 2010) and positive attitudes focusing on others.

Different prosocial behaviors are not always correlated with each other (e.g., Bryant and Crockenberg, 1980; Knafo et al., 2011b). Similarly, prosocial attitudes are associated with prosocial behaviors only under certain situational conditions (e.g., Anker et al., 2010). Nevertheless, when different prosocial behaviors are aggregated, they do relate substantially to prosocial values (e.g., Bardi and Schwartz, 2003), and there is empirical evidence that empathy is associated with prosocial behavior (Knafo et al., 2008). These inconsistent or conditional associations fueled a debate on the possibility of an “altruistic personality” or a “prosocial personality” in past research (Eisenberg et al., 1999; Penner et al., 2005; Knafo and Israel, 2012). The first goal of the current research is to contribute to this debate by studying the structure of a broad set of attitudinal, behavioral, and affective of children’s prosociality.

The second goal of this paper is to understand the nature of the associations among different aspects of prosociality. At the evolutionary level, it has been suggested that empathy evolved in humans as a necessity for group living and taking care of infants, thus facilitating different aspects of prosocial behavior (de Waal, 2008). Social psychological research suggests that manipulation of empathy levels, such as activating empathy experimentally, increases the likelihood of prosocial behaviors including helping and sharing (Van Lange, 2008; Batson, 2010). These contexts account for empathy and prosocial behavior at the mean level. However, less is known about the association between empathy and prosocial behavior at the level of the individual, i.e., some individuals are more empathic *and* are more prosocial than others. Such an association supports a “prosocial personality” view.

The third goal of the current research is to investigate the environmental and genetic effects on prosociality. Socialization research typically finds that the parenting correlates for empathy/sympathy and prosocial behavior overlap. Responsive and accepting parenting, which may enhance a sense of connection to others, as well as exposure to prosocial models, have been related to both children’s sympathy and prosocial behaviors (see Eisenberg et al., 2015, for review). Thus, the same environmental influences may account for the association between different facets of prosociality.

Genetic effects may also contribute to individual differences in prosociality. There is substantial evidence for the heritability of prosocial behavior and empathy (see Knafo and Israel, 2009 and Fortuna and Knafo, 2014 for reviews; Knafo and Uzefovsky, 2013, for meta-analysis on empathy). Similarly, prosocial values and attitudes show substantial genetic influences (Rushton, 2004; Knafo and Spinath, 2011). Genetics have been shown to account for the consistency of prosocial attitudes across social domains (Lewis and Bates, 2011), yet evidence linking empathy to prosocial behavior and to prosocial attitudes at the genetic level is sparse.

One study using adults’ self-reports found that individual differences in helpfulness and compassion had shared genetic origins. They also had common environmental origins, overlapping with empathy, which was not heritable in this particular study (Ando et al., 2004). Another study found inconsistent evidence for the role of genetics and the shared environment in the association between children’s observed empathy and prosocial behaviors (Knafo et al., 2008). In summary, more research is needed to determine whether the same genetic and environmental factors apply to the different prosociality facets.

The current investigation studied the structure of prosociality and the underlying genetic and environmental contributions to this structure using maternal reports of children’s empathy, prosocial behavior, and prosocial attitudes. In order to ensure that congruency and differences across items and scales would not be confounded by differences across reporter sources, all data presented here are based on mother reports. Mother reports of prosociality have been shown in previous research to have substantial validity (Zahn-Waxler and Radke-Yarrow, 1982; Zahn-Waxler et al., 1992). Moderate associations were found between mother reports and the reports of fathers, teachers, and the child himself or herself (Davé et al., 2008; Howe et al., 2008; Malti et al., 2009). In addition, parent reports on empathy and prosocial behavior have been linked to observed or experimentally induced behaviors (Dadds et al., 2008; Ensor et al., 2011). In this report we chose to focus on mother reports because we were interested in the factor structure as well as the genetic and environmental structure of prosociality. Relying on a single reporter enables having a single source of error variance rather than different sources associated with different measures. This enables a clearer interpretation of congruence and difference across different items and scales.

We addressed the role of genetics and the environment with the twin design, a widely used method (Plomin et al., 2001). This design compares twin similarity for a given phenotype across pairs of monozygotic (MZ) and dizygotic (DZ) twins. Because MZ twins are virtually 100% genetically identical, while DZ twins share on average half of their genetic sequence, greater similarity of MZ as compared to DZ twins indicates genetic influence (*heritability*). When twins are more similar to each other than would be expected based on the genetic effect (i.e., when DZ twin similarity is higher than about half of the MZ similarity), this similarity is attributed to the environment that twins have in common (*shared environment* effect). Finally, any further differences between twins are attributed to *non-shared environment* and measurement errors independent across the two twins.

To summarize our approach, we first looked for the presence of common facets of prosociality. We expected to find facets that would represent the affective aspect of prosociality (empathy), the attitudinal aspect (prosocial attitude/kindness), and the behavioral aspect, specifically the three most common behaviors of sharing, helping, and comforting. We then tested whether a common factor accounted for the variance in these facets of prosociality. Finally, following past research, we expected to observe both genetic and environmental contributions to individual differences in prosociality. Using a multivariate genetic design, we studied the environmental and genetic contributions to the common prosociality factor as well as to the different facets of prosociality.

## MATERIALS AND METHODS

### PARTICIPANTS

Families in this study were participants in the Longitudinal Israeli Study of Twins (LIST). In this study of social development, parents of all Hebrew-speaking families of twins born in Israel during 2004–2005 were invited to participate through parent questionnaires when the twins reached the age of 3 (Knafo, 2006). At age 7, recruitment was done only for lab visits, reducing the number of participating families and concentrating on families from the greater Jerusalem area. See Avinun and Knafo (2013) for details on recruitment and representativeness of the sample.

The average age of the sample was 90 months (SD = 3.85). The sex distribution was about equal (51.6% males). The sample included 57 MZ, 108 DZ same-sex, and 18 opposite-sex twin pairs. (The latter group was under-represented because of budgetary considerations. We used their data in the descriptive, but not in the genetic analyses.)

### PROCEDURE

When the twins reached the age of 3 years, and again when they were 5-years-old, mothers filled out mailed questionnaires which included questions on the twin’s behavior and development, demographic details, and questions regarding the twins’ zygosity. At age 7, families living within 1 h drive from the Jerusalem lab were invited to the lab for assessment. While twins participated in a series of tasks beyond the scope of the current paper, mothers were given questionnaires about children’s development, which included prosociality scales. The study was approved by the Hebrew University Social Sciences research ethics committee.

### MEASURES

*Twin zygosity* was assessed using information from DNA samples. When that was not available, we used an algorithm calculated according to a parental questionnaire of physical similarity (Goldsmith, 1991), which is in agreement with the DNA testing in over 95% of the cases.

**FIGURE 1:**
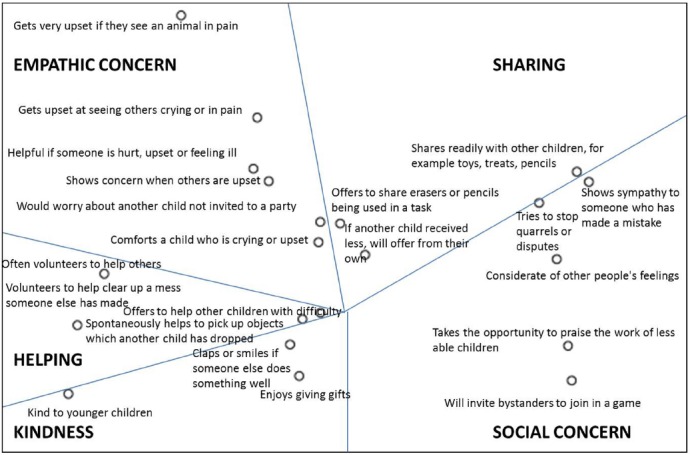
**Multidimensional scaling of the prosociality items.** Item labels are abbreviated from the original items, for presentation purposes. Stress = 0.258, *R*^2^ = 0.66.

*Prosociality scales*. Mothers rated the behavior of each of their children with a total of 21 items taken from three main sources (abbreviated items appear in Figure 1). As in previous waves of LIST (e.g., Knafo et al., 2011a) we used the prosocial behavior subscale (five items) of the Strengths and Difficulties Questionnaire (SDQ; Goodman, 1997). An exemplary item is “Often volunteers to help others (parents, teachers, other children).”

In addition, we used items from the Prosocial Behavior Questionnaire (PBQ; Weir and Duveen, 1981). Of the 20 original items, we excluded items that focused on compliance to social rules, and those greatly overlapping with SDQ items, retaining 10 items that specifically measured prosocial behavior (items 1, 2, 3, 9, 10, 11, 12, 15, 18, 19). Importantly, several items were slightly adapted from the original version which was aimed for teachers (e.g., dropped reference to classroom). Example items are: “Shows sympathy to someone who has made a mistake” and “Comforts a child who is crying or upset.”

To address empathy, we used four items from Baron–Cohen’s Empathy Quotient (EQ; Auyeung et al., 2009). Items were selected to refer specifically to empathy and not to prosocial behavior (items 31, 37, 45, 48 in the original questionnaire). An example item is “Gets upset at seeing others crying or in pain.”

Two more items were added for the purpose of the current investigation (“Enjoys giving gifts to adults or other children” and “If he/she sees that another child received less, will offer from their own”).

Items were interspersed among other (non-prosocial) SDQ items. Mothers rated children’s behavior with the SDQ scale, indicating whether various symptoms and behaviors are “Not True,” “Somewhat True” or “Certainly True” of their child (scored, respectively, as 0, 1, or 2).

## RESULTS

### IDENTIFYING FACETS OF PROSOCIALITY

To understand the structure of prosociality, we ran a multidimensional scaling (MDS) analysis on the 21 items. MDS is part of a family of methods used to represent constructs in a space, in order to understand the structure of a construct based on the relative location in the space (e.g., Schwartz, 1992). Items are seen as sampled from a continuum (content universe; Shye, 1998) of possible items. Interpretation of the structure is based on theoretical considerations and face validity and content meaningfulness of the groups of items (e.g., Daniel et al., 2013).

We used the alternating least-squares algorithm (ALSCAL) procedure in SPSS 20, adding no initial configuration (Borg and Groenen, 2010). Figure 1 presents the item configuration of the MDS. The closer items are in the space, the more they have a shared meaning. We attempted to find meaningful regions in the MDS configuration, based on our initial consideration of empathy, prosocial attitudes, and three kinds of prosocial behaviors (sharing, helping, and comforting). This process identified five meaningful clusters of at least three items each (Figure 1). Importantly, each of the regions included items from at least two of the original questionnaires we have used (SDQ, EQ, and PBQ), indicating that items were organized based more on content and less on questionnaire of origin. We describe each of these regions below, starting from the upper-left-hand corner of the figure and moving clockwise.

The first facet includes six items describing children’s reaction to others in distress. Although we initially expected to find a specific region of comforting behavior, such items appeared together with items tapping the affectionate reactions of empathy. We labeled this facet *empathic concern*.

The second facet included three items of *sharing*, or giving resources to others. Next, all items representing the attitudinal aspect of prosociality appeared together. Nevertheless, these items showed a clear distinction between two groups of items: *social concern* (five items), including behaviors focused on making others feel better and improving social relationships (without a focus on others’ distress as in the empathic concern items), and *kindness* (three items), reflecting a positive outlook on others, including sharing in others’ positive affect. Notably, these last two regions largely correspond to a prosocial attitude, although social concern includes also behavioral items, and kindness focuses mainly on positive feelings towards others. We therefore retained them as separate constructs. Finally, the last region included four items of instrumental *helping*.

We averaged the items in each region (alphas ranging from 0.61 to 0.79) to form scores on each of the five facets of prosociality.

### DESCRIPTIVES OF THE PROSOCIALITY FACETS

Table 1 presents the means and standard deviations of the five prosociality facets in girls and boys. Preliminary analyses found no mean differences between MZ and DZ twins or between firstborn and second-born twins, nor did these variables interact with gender.

**Table 1:** **Means and standard deviations of scores on the prosociality facets**.

	**Boys**		**Girls**		**Sex difference**	
	**Mean**	**SD**	**Mean**	**SD**	**t**	**D**
Sharing	1.20	0.51	1.38	0.53	3.11	0.33
Social concern	1.03	0.39	1.19	0.43	2.85	0.30
Kindness	1.42	0.48	1.61	0.38	3.48	0.36
Helping	1.05	0.48	1.24	0.45	4.16	0.44
Empathic concern	1.27	0.44	1.51	0.38	3.92	0.41

All sex differences significant, p < 0.005.

Descriptive analyses were conducted in Mplus (Muthén and Muthén, 1998-2007). Twins were clustered within twin pairs, with standard errors computed using the TYPE = COMPLEX option, taking into account the fact that twin-data are non-independent of each other. These analyses showed that in all facets girls scored higher than boys (Table 1), with small to moderate effect sizes (*D* = 0.30–0.44). This replicates results from previous research (Eisenberg et al., 2006).

Interestingly, all prosociality factors were substantially intercorrelated, with correlations ranging from 0.39 (between social concern and kindness) and 0.57 (between social concern and sharing; see Table 2). These appreciable intercorrelations, taken together with the theoretical idea that a single prosociality factor exists, prompted us to investigate the shared origins of the five facets.

**Table 2:** **Intercorrelations among prosociality facets**.

	**Social concern**	**Kindness**	**Helping**	**Empathic concern**
Sharing	0.57	0.43	0.50	0.56
Social concern		0.39	0.47	0.53
Kindness			0.46	0.44
Helping				0.52

All correlations significant, p < 0.001.

### FACTOR STRUCTURE OF THE PROSOCIALITY FACETS

As stated above, it has been suggested that a single prosociality tendency explains different types of prosocial behaviors. Therefore we conducted a confirmatory factor analysis (CFA) in which all prosociality facets loaded on a single factor. The model had an excellent fit to the data, χ^2^(df = 55) = 5.49, *ns*, comparative fit index (CFI) = 0.999, root-mean-square error of approximation (RMSEA) = 0.016. Loadings ranged from 0.59 to 0.76. Thus, results supported a single-factor solution. Nevertheless, the factor only accounted for 35–57% of the variance in each of the facets, suggesting that each facet has additional unique variance, as is also indicated by the size of the correlations in Table 2. In the genetic analyses below, we tried to understand the sources of these shared and unique variances.

### GENETIC AND ENVIRONMENTAL EFFECTS ON THE PROSOCIALITY FACETS

To examine genetic and environmental influences on the prosociality facets, we began by comparing MZ and DZ twin correlations for each of the prosociality facets (shown in boldface type in Table 3). Because of the sex differences reported above, and because the sample size was not sufficient for studying sex-limitation models of the genetic/environmental contributions to prosociality (e.g., Knafo and Plomin, 2006), we only used data from same-sex twins, Z-standardized separately for girls and boys in the genetic analyses.

**Table 3:** **Twin correlations on the prosociality facets**.

	**Sharing**	**Social concern**	**Kindness**	**Helping**	**Empathic concern**
MZ twins					
Sharing	0.72**	0.39**	0.29*	0.29*	0.37**
Social concern	0.38**	0.63**	0.11	0.08	0.31*
Kindness	0.31*	0.13	0.81**	0.39**	0.15
Helping	0.31*	0.31*	0.24	0.54**	0.09
Empathic concern	0.32*	0.38**	0.22	0.05	0.78**
DZ twins					
Sharing	0.19*	0.01	0.10	0.06	0.08
Social concern	–0.04	**0.17**	0.12	–0.03	–0.12
Kindness	0.11	0.17	0.60**	0.11	0.10
Helping	0.03	–0.03	0.23*	**0.01**	–0.07
Empathic concern	0.05	0.04	0.16	0.06	0.20*

Rows represent the prosociality scores of one twin, and columns represent the scores of his or her co-twin. *p ;< 0.05, ** p < 0.01.

For all prosociality facets, MZ correlations were larger than DZ correlations, indicating genetic influence (see Table 3). MZ correlations were, of course, less than 1.0, indicating the additional influence of non-shared environment and measurement error. For *kindness* specifically, there was an indication for a shared environmental influence, as the DZ correlation was greater than half the MZ correlation, indicating non-genetic contribution to twin similarity.

To examine genetic and environmental effects more directly, we used model-fitting in the Mx structural equation modeling software (Neale et al., 1999). As observed from the correlations, a genetic effect was detected for all prosociality facets (Table 4), accounting for 44–76% of the variance, across facets. For four of the facets, the contribution of the shared environment effect was estimated at zero and could be dropped from the model, except for the case of kindness, where the shared environment effect was estimated at 35%. Dropping it would result in a worse model fit, χ^2^(df = 1) = 6.19, *p* < 0.02, and it was thus retained in the model. Finally, for all variables a meaningful (18–56%) non-shared environment effect (which also includes measurement error) was estimated.

**Table 4:** **Estimates of variance components (and 95% confidence intervals) accounting for individual differences in prosociality facets**.

**Prosociality facets**	**Heritability**	**Shared environment**	**Non-shared environment and error**
Sharing	0.67 (0.5–0.78)	0.00 (0.00–0.00)	0.33 (0.22–0.49)
Social concern	0.56 (0.37–0.70)	0.00 (0.00–0.00)	0.44 (0.30–0.63)
Kindness Helping Empathic concern	0.47 (0.22–0.75)	0.35 (0.08–0.56)	0.18 (0.12–0.27)
Helping	0.44 (0.18–0.64)	0.00 (0.00–0.00)	0.56 (0.36–0.82)
Empathic concern	0.76 (0.62–0.85)	0.00 (0.00–0.00)	0.24 (0.15–0.38)

Heritability estimated as an additive genetic effect.

As reported above, a single factor underlies the association between the different prosociality facets. Could this common factor be explained by shared genetic and environmental influences? One indication that common genetic effects account for the variance common to the different facets can be seen in the cross-twin cross-trait correlations (Table 3). For example, one twin’s kindness correlates with the co-twin’s helping substantially in MZ pairs (0.39), but only weakly in DZ pairs (0.11).

We used the Common-Factor-Common-Pathways Multivariate model (e.g., Rijsdijk and Sham, 2002) to investigate the genetic and environmental contributions common and unique to the different facets. This model assumes that there is an underlying common factor accounting for individual differences across the different prosociality facets, and provides an estimate of the proportion of variance in each facet associated with the common factor. In addition, the model estimates the remaining, residual variance that is unique to each facet that is not accounted for by the common factor. The benefit of this model is that it can be used to disentangle genetic and environmental effects unique to each facet from those applying across the prosociality facets.

**FIGURE 2:**
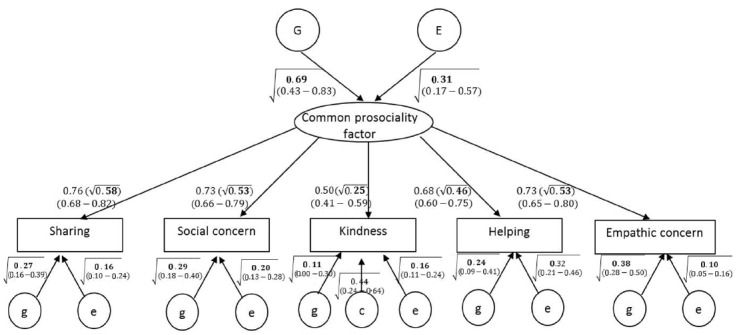
**Result of common-factor-common-pathways multivariate model of genetic and environmental effects on prosociality.** Rectangles indicate observed scores on prosociality facets. Rounded shapes indicate the common prosociality factor and the variance components estimates. G = heritability and E = non-shared environment (and error) contributions to the common factor. For each observed score unique variance components are also estimated, for which g = genetic; c = shared environment; e = non-shared environment (and error) contributions to the unique variance of each observed score. Values in parentheses are 95% confidence intervals. Values within the square root sign are squared standardized paths and represent the percentage of variance accounted for by the variance component. Values on paths from the common factor to the observed score represent loading of the latent common factor on the observed score (values within the square root sign indicate the proportion of the variance accounted for by the latent common factor).

One factor affecting all prosociality facets is estimated. The magnitudes of genetic influences, shared environmental influences, and non-shared environmental influences are estimated for this common prosociality factor. In addition, the model estimates the magnitude of variable-specific genetic and environmental influences. Initially, we tested a full model, but as the shared environment effects on the common factor and all the prosociality facets except kindness were estimated at zero (see also Table 4), we dropped them from the model, which did not worsen model fit, Δχ^2^(df = 5) = 2.94, *ns*. We present the results of the reduced model in Figure 2.

#### The underlying common prosociality factor

The five prosociality facets loaded positively on the underlying common prosociality factor, with standardized loadings ranging from 0.50 to 0.76. The squared standardized loadings are equal to the proportion of the variance in each prosociality facet accounted for by the latent common factor. Each facet also had unique variability not accounted for by the common prosociality factor. This echoes the previous finding of a single common prosociality factor reported above.

#### Genetic and environmental influences on the common prosociality factor

The upper part of Figure 2 presents the estimates and 95% confidence intervals of the variance components accounting for individual differences in the common prosociality factor. (The square root symbol indicates that numbers in the figure are squared standardized paths, representing proportion of variance.) Consistent with the MZ and DZ correlations and with the results of the univariate genetic models (Table 4), a strong (69%) genetic effect emerged for the common factor. The remaining variance in the common factor was accounted for by non-shared environment (and any measurement error that is common to the five prosociality facets).

#### Genetic and environmental influences on the unique prosociality components

Figure 2 also presents, for each prosociality facet separately, the genetic and environmental contributions to the variance not accounted for by the common factor. For example, in sharing, the common factor accounts for 58% of the variability (0.76^2^), with the remaining 42% roughly equal (due to rounding error) to the summed effects of an additional, unique genetic effect (27%) and a unique non-shared environment effect and measurement error (16%).

Unique genetic effects were observed for all of the prosociality facets, although the effect on kindness was not significant, as indicated by the confidence intervals. In kindness, a unique shared environment effect (44%) emerged. Finally, in addition to the non-shared environment effects on the common factor, unique non-shared environment effects (10–32%) were found for all prosociality facets. These effects include the measurement error unique to each facet.

#### Non-additive genetic effects

Except for the case of kindness, the MZ correlations were substantially higher than twice the DZ correlations (Table 2), raising the possibility of non-additive genetic effects (shared by MZ twins at a 100%, but by DZ twins at an average of 25%). The modest size of our sample limited our ability to detect non-additive effects. Therefore, the following analyses regarding such effects are suggestive.

It is not possible to test for both non-additive and shared environment effects in the same model (because both are estimated by comparing MZ to DZ twins). In addition, the correlation pattern for kindness did not indicate any non-additive genetic effect. Therefore, a non-additive genetic effect was not tested for kindness. In the univariate analyses for sharing and social concern, either the additive or the non-additive genetic effect could be dropped without affecting model fit. In two other facets, dropping the non-additive effect resulted in worsening fit (helping, χ^2^(df = 1) = 6.94, *p* < 0.01; empathic concern, χ^2^(df = 1) = 6.19, *p* < 0.02), indicating that the genetic effect was, at least in part, of the non-additive kind.

We next estimated the Common-Factor-Common-Pathways multivariate model, extending it to include non-additive genetic effects as unique genetic components for all facets except kindness. The additive unique genetic effect on helping, as well as the non-additive genetic effects on sharing, social concern, and empathic concern were all estimated at zero or close to zero, and were dropped from the model together with the shared environment effects without affecting model fit, χ^2^(df = 10) = 3.11, *ns*. The final model was very similar to the one presented in Figure 2, except for the estimation of the unique genetic effect on helping as non-additive.

## DISCUSSION

Is prosociality a single construct, or is it a multifaceted trait? After combining items from prosociality scales covering affective, behavioral, and attitudinal measures, we find that a single factor of prosociality accounts for a substantial proportion of the variance across all prosociality facets, as reported by mothers. This empirical evidence is consistent with the theoretical conceptualization of a prosocial “personality.”

The current results also provided an account of a common genetic factor relevant to all studied facets of prosociality. More research is needed to understand how this common genetic factor operates. A recent innovative study found that variation in the oxytocin receptor gene indirectly predicted prosocial behavior through empathic concern (Christ et al., 2015). Thus, in this account genetics affect prosocial behavior through empathy. This is consistent with the idea that empathy as a trait motivates prosocial behavior (Batson, 2010), and with the early emergence of empathy (Davidov et al., 2013). However, longitudinal research is needed to understand whether indeed an initial genetic predisposition for empathy is generalized to a genetic tendency for prosocial behavior across development.

Although a key conclusion of our research is that a single prosociality factor largely accounts for different prosociality facets as reported by mothers, at least 40% of the variance for each facet was unaccounted for by the common factor. Moreover, each of the facets described a distinct conceptual domain reflecting its focus on behavior or affect, or on the nature of the behavior it measured. This pattern of findings demonstrates the complexity of the prosociality construct (see Padilla-Walker and Carlo, 2014). For example, sharing and helping, both prosocial behaviors by virtue of focusing on the benefit of others, only showed a correlation of 0.50 in this study. Whether research focuses on the specific facets or on the common prosociality factor should depend on the degree of interest in the specificity of each facet.

Future research should study in further depth the complexity of prosociality (for review, see Eisenberg et al., 2015). For example, prosocial behaviors may be self-initiated (i.e., spontaneous, performed without an explicit request) or compliant (performed in response to an explicit request; Knafo et al., 2011b). Similarly, different prosocial acts may reflect different motivations (Eisenberg and Spinrad, 2014). Finally, future accounts of the prosocial personality should refer to the possibility that individual differences in prosociality are situation-dependent, as some children are consistently highly prosocial, while others’ prosociality may vary depending on the situational contingencies (Mischel and Shoda, 1995).

In addition to the common factor, for each facet (except kindness), unique genetic and environmental contributions were identified as well. The meaning of this finding is that different developmental forces operate on the different facets. For example, in some families parents may encourage prosocial behaviors but not necessarily empathy as the latter often includes a negative affective reaction, leading to a differentiation among the different prosociality facets. The unique genetic effects may relate to the specific social and situational aspects of each facet. For example, shy temperament may interfere with children’s helping others, but should not affect their empathy. In addition, for helping and possibly also empathic concern, evidence for a non-additive genetic effect suggests a different form of genetic transmission than the other facets. In future research, it would be important to further our understanding of genetic effects by looking at specific genetic polymorphisms (e.g., Ebstein et al., 2010), as they relate to both the common factor and its constituent facets.

The finding of small or null shared environment effects for most prosociality facets replicates the results of past research. This research found evidence for weak or absent shared environment effects beyond age 3, using parent, self, and teacher reports (e.g., Knafo and Plomin, 2006; Gregory et al., 2009; Knafo and Israel, 2009), as well as observational and experimental measures (Knafo et al., 2008, 2011a; for an exception see van IJzendoorn et al., 2010). As discussed by Knafo and Plomin (2006), this pattern may seem at odds with evidence for the role of parenting in prosociality (e.g., Davidov and Grusec, 2006). However, children are increasingly exposed to additional, non-familial environments, which can increase sibling differences. In addition, parenting–prosociality associations may reflect cases in which parenting behavior occurs in reaction to the child’s genetically influenced behavior. Such gene–environment correlations appear as part of the heritability estimate, because they are driven by genetic differences between the siblings (e.g., Knafo and Jaffee, 2013). Parenting differences between the twins (i.e., differential treatment) that are not driven by the child’s genotype can also affect behavior, and may be expressed in the non-shared environment estimates. Finally, and importantly, the same parenting effect can yield different developmental outcomes if such socialization is directed at genetically different siblings, such as DZ twins. While it is beyond the scope of the present research, it is important for future research to also investigate such gene–environment interactions (e.g., Knafo et al., 2011a).

The genetic analyses showed evidence for shared environment effects only for the kindness variable. Future research, preferably with more elaborate scales for kindness, should seek to replicate this finding and understand why it is this specific facet that shows a shared environment effect. For example, family-wide variables such as religiosity and socioeconomic status could be introduced to the twin design to help understand the role of the shared environment in kindness and in the other facets.

Importantly, meaningful non-shared environment effects were found for the global prosociality factor as well as for all facets. To address the effects of the non-shared environment, developmental predictors (e.g., medical history or life events) unique to each child could be investigated with regards to twin differences in prosociality. Within a MZ twin design, such behavioral differences would be attributed mainly to the environment (or to its interaction with genes) and not to the genetic differences between twins.

The limitations of this study include a modest sample size for a twin study, which did not allow for in-depth-examination of sex-limitation models or inclusion of measured environmental effects. In addition, while mother reports are a common, valid, and useful tool for measuring child behavior, there is the possibility of common method variance accounting in part for the associations across facets of prosociality. Future research would also benefit from complimentary methods, such as experimentally derived or naturally observed child behaviors. In our ongoing longitudinal study we have been collecting relevant data (e.g., Knafo et al., 2011a), which we will be able to use in the future to address our questions. In spite of these limitations, this study provides a unique treatment of the prosocial personality question, covering individual differences in a variety of prosociality facets and studying their joint and separate genetic and environmental origins, opening future paths for understanding this noble aspect of human nature.

### Conflict of Interest Statement

The authors declare that the research was conducted in the absence of any commercial or financial relationships that could be construed as a potential conflict of interest.

## References

[B1] Ando J. Suzuki A. Yamagata S. Kijima N. Maekawa H. Ono Y. (2004). Genetic and environmental structure of Cloninger’s temperament and character dimensions. J. Pers. Disord. 18, 379–393. 15342324 10.1521/pedi.18.4.379.40345

[B2] Anker A. E. Feeley T. H. Kim H. (2010). Examining the attitude–behavior relationship in prosocial donation domains. J. Appl. Soc. Psychol. 40, 1293–1324 10.1111/j.1559-1816.2010.00619.x

[B3] Auyeung B. Wheelwright S. Allison C. Atkinson M. Samarawickrema N. Baron-Cohen S. (2009). The children’s empathy quotient and systemizing quotient: sex differences in typical development and in autism spectrum conditions. J. Autism Dev. Disord. 39, 1509–1521. 10.1007/s10803-009-0772-x 19533317

[B4] Avinun R. Knafo A. (2013). The Longitudinal Israeli Study of Twins (LIST) - an integrative view of social development. Twin Res. Hum. Genet. 16. 10.1017/thg.2012.73 23394191

[B5] Bardi A. Schwartz S. H. (2003). Values and behavior: strength and structure of relations. Pers. Soc. Psychol. Bull. 29, 1207–1220. 10.1177/0146167203254602 15189583

[B6] Batson C. D. (2010). “Empathy-induced altruistic motivation,” in Prosocial Motives, Emotions, and Behavior, eds Shaver P. R. Mikulincer M. (Washington, DC: American Psychological Association Publications), 15–34.

[B7] Borg I. Groenen P. J. F. (2010). Modern Multidimensional Scaling: Theory and Applications. Secaucus, NJ: Springer.

[B8] Bryant B. K. Crockenberg S. B. (1980). Correlates and dimensions of prosocial behavior: a study of female siblings with their mothers. Child Dev. 52, 9–544. 10.2307/1129288 7398455

[B9] Christ C. Carlo G. Stoltenberg S. (2015). Oxytocin receptor (OXTR) single nucleotide polymorphisms indirectly predict prosocial behavior through perspective taking and empathic concern. J. Pers. 10.1111/jopy.12152 [Epub ahead of print]. 25403479

[B10] Dadds M. R. Hunter K. Hawes D. J. Frost A. D. Vassallo S. Bunn P. (2008). A measure of cognitive and affective empathy in children using parent ratings. Child Psychiatry Hum. Dev. 39, 111–122. 10.1007/s10578-007-0075-4 17710538

[B11] Daniel E. Hoffman-Towfigh N. Knafo A. (2013). School values: a new typology of school value dimensions across three cultures. SAGE Open 3. 10.1177/2158244013482469

[B12] Davé S. Nazareth I. Senior R. Sherr L. (2008). A comparison of father and mother report of child behaviour on the Strengths and Difficulties Questionnaire. Child Psychiatry Hum. Dev. 39, 399–413. 10.1007/s10578-008-0097-6 18266104

[B13] Davidov M. Grusec J. E. (2006). Untangling the links of parental responsiveness to distress and warmth to child outcomes. Child Dev. 77, 44–58. 10.1111/j.1467-8624.2006.00855.x 16460524

[B14] Davidov M. Zahn-Waxler C. Roth-Hanania R. Knafo A. (2013). Concern for others in the first year of life: theory, evidence, and future directions. Child Dev. Perspect. 7, 126–131 10.1111/cdep.12028

[B15] de Waal F. B. M. (2008). Putting the altruism back into altruism: the evolution of empathy. Annu. Rev. Psychol. 59, 279–300. 10.1146/annurev.psych.59.103006.093625 17550343

[B16] Ebstein R. P. Israel S. Chew S. H. Zhong S. Knafo A. (2010). Genetics of human social behavior. Neuron 65, 831–844. 20346758 10.1016/j.neuron.2010.02.020

[B17] Eisenberg N. Fabes R. A. Spinrad T. (2006). “Prosocial development,” in Handbook of Child Psychology, Vol. 3, *Social, Emotional, and Personality Development*, 6th Edn, Vol. ed. Eisenberg N.; Series eds Damon W. Lerner R. M. (New York: Wiley), 646–718.

[B18] Eisenberg N. Guthrie I. K. Murphy B. C. Shepard S. A. Cumberland A. Carlo G. (1999). Consistency and development of prosocial dispositions: a longitudinal study. Child Dev. 70, 1360–1372. 10621961 10.1111/1467-8624.00100

[B19] Eisenberg N. Spinrad T. L. (2014). “Multidimensionality of prosocial behavior,” in The Complexities of Raising Prosocial Children: An Examination of the Multidimensionality of Prosocial Behaviors, eds Padilla-Walker L. Carlo G. (Oxford: Oxford University Press), 17–39.

[B20] Eisenberg N. Spinrad T. L. Knafo-Noam A. (2015). “Prosocial development,” in Handbook of Child Psychology, Vol. 3, *Social, Emotional, and Personality Development,* 7th Edn, Vol. eds Lamb M. E. Garcia Coll C.; Series ed. Lerner R. M. (New York: Wiley), 610–656.

[B21] Ensor R. Spencer D. Hughes C. (2011). ‘You feel sad?’ Emotion understanding mediates effects of verbal ability and mother–child mutuality on prosocial behaviors: findings from 2 years to 4 years. Soc. Dev. 20, 93–110 10.1111/j.1467-9507.2009.00572.x

[B22] Fortuna K. Knafo A. (2014). “Parental and genetic contributions to prosocial behavior during childhood,” in The Complexities of Raising Prosocial Children: An Examination of the Multidimensionality of Prosocial Behaviors, eds Padilla-Walker L. Carlo G. (Oxford University Press), 70–89.

[B23] Goldsmith H. H. (1991). A zygosity questionnaire for young twins: a research note. Behav. Genet. 21, 257–269. 1863259 10.1007/BF01065819

[B24] Goodman R. (1997). The strengths and difficulties questionnaire: a research note. J. Child Psychol. Psychiatry 38, 581–586. 9255702 10.1111/j.1469-7610.1997.tb01545.x

[B25] Gregory A. M. Light-Häusermann J. H. Rijsdijk F. Eley T. C. (2009). Behavioral genetic analyses of prosocial behavior in adolescents. Dev. Sci. 12, 165–174. 10.1111/j.1467-7687.2008.00739.x 19120424

[B26] Howe A. Pit-ten Cate I. M. Brown A. Hadwin J. A. (2008). Empathy in preschool children: the development of the Southampton Test of Empathy for Preschoolers (STEP). Psychol. Assess. 20, 305. 10.1037/a0012763 18778167

[B27] Knafo A. (2006). The Longitudinal Israeli Study of Twins (LIST): children’s social development as influenced by genetics, abilities, and socialization. Twin Res. Hum. Genet. 9, 791–798. 10.1375/twin.9.6.791 17254410

[B28] Knafo A. Israel S. (2009). “Genetic and environmental influences on prosocial behavior,” in Prosocial Motives, Emotions, and Behavior: The Better Angels of Our Nature, eds Mikulincer M. Shaver P. R. (Washington, DC: American Psychological Association Publications), 149–167.

[B29] Knafo A. Israel S. (2012). “Empathy, prosociality, and other aspects of kindness,” in The Handbook of Temperament: Theory and Research, eds Zentner M. Shiner R. (New York: Guilford Press), 168–179.

[B30] Knafo A. Israel S. Ebstein R. P. (2011a). Heritability of children’s prosocial behavior and differential susceptibility to parenting by variation in the dopamine D4 receptor (DRD4) gene. Dev. Psychopathol. 23, 53–67. 10.1017/S0954579410000647 21262039

[B31] Knafo A. Steinberg T. Goldner I. (2011b). Children’s low affective perspective-taking ability is associated with low self-initiated prosociality. Emotion 11, 194–198. 10.1037/a0021240 21401240

[B32] Knafo A. Jaffee S. (2013). Gene–environment correlations in developmental psychopathology. Dev. Psychopathol. 25 10.1017/S0954579412000855 23398748

[B33] Knafo A. Plomin R. (2006). Prosocial behavior from early to middle childhood: genetic and environmental influences on stability and change. Dev. Psychol. 42, 771–786. 10.1037/0012-1649.42.5.771 16953685

[B34] Knafo A. Spinath F. M. (2011). Genetic and environmental influences on girls’ and boys’ gender-typed and gender-neutral values. Dev. Psychol. 47, 726–731. 10.1037/a0021910 21142356

[B35] Knafo A. Uzefovsky F. (2013). “Variation in empathy: the interplay of genetic and environmental factors,” in The Infant Mind: Origins of the Social Brain, eds Legerstee M. Haley D. W. Bornstein M. H. (New York: Guilford Press), 97–122.

[B36] Knafo A. Zahn-Waxler C. Van Hulle C. Robinson J. Rhee S. H. (2008). The developmental origins of a disposition toward empathy: genetic and environmental contributions. Emotion 8, 737–752. 10.1037/a0014179 19102585

[B37] Lewis G. J. Bates T. C. (2011). A common heritable factor influences prosocial obligations across multiple domains. Biol. Lett. rsbl20101187 10.1098/rsbl.2010.1187 PMC3130221 21307044

[B38] Malti T. Gummerum M. Keller M. Buchmann M. (2009). Children’s moral motivation, sympathy, and prosocial behavior. Child Dev. 80, 442–460. 10.1111/j.1467-8624.2009.01271.x 19467003

[B39] Mischel W. Shoda Y. (1995). A cognitive-affective system theory of personality: reconceptualizing situations, dispositions, dynamics, and invariance in personality structure. Psychol. Rev. 102, 246. 7740090 10.1037/0033-295x.102.2.246

[B40] Muthén, L. K., and Muthén, B. O. (1998-2007). *Mplus. Statistical Analyses With Latent Variables* *User’s Guide, 3*.

[B41] Neale M. C. Boker S. M. Xie G. Maes H. M. (1999). Statistical Modeling. Richmond, VA: Department of Psychiatry.

[B42] Nowak M. A. (2006). Five rules for the evolution of cooperation. Science 314, 1560–1563. 10.1126/science.1133755 17158317 PMC3279745

[B43] Padilla-Walker L. M. Carlo G. (2014). Prosocial Development: A Multidimensional Approach. Oxford: Oxford University Press.

[B44] Penner L. A. Dovidio J. F. Piliavin J. A. Schroeder D. A. (2005). Prosocial behavior: multilevel perspectives. Annu. Rev. Psychol. 56, 365–392. 10.1146/annurev.psych.56.091103.070141 15709940

[B45] Plomin R. DeFries J. C. McClearn G. E. McGuffin P. (2001). Behavioral Genetics, 4th Edn. New York: Worth Publishers.

[B46] Rushton J. P. (2004). Genetic and environmental contributions to pro-social attitudes: a twin study of social responsibility. Proc. R. Soc. Lond. B, Biol. Sci. 271, 2583–2585. 10.1098/rspb.2004.2941 15615684 PMC1691905

[B47] Rijsdijk F. V. Sham P. C. (2002). Analytic approaches to twin data using structural equation models. Brief. Bioinform. 3, 119–133. 10.1093/bib/3.2.119 12139432

[B48] Schwartz S. H. (1992). Universals in the content and structure of values: theoretical advances and empirical tests in 20 countries. Adv. Exp. Soc. Psychol. 25, 1–65.

[B49] Schwartz S. H. (2010). “Basic values: how they motivate and inhibit prosocial behavior,” in Prosocial Motives, Emotions, and Behavior, eds Shaver P. R. Mikulincer M. (Washington, DC: American Psychological Association Publications), 221–241.

[B50] Shye S. (1998). Modern facet theory: content design and measurement in behavioral research. Eur. J. Psychol. Assess. 14, 160.

[B51] van IJzendoorn M. H. Bakermans-Kranenburg M. J. Pannebakker F. Out D. (2010). In defence of situational morality: genetic, dispositional and situational determinants of children’s donating to charity. J. Moral Educ. 39, 1–20 10.1080/03057240903528535

[B52] Van Lange P. A. M. (2008). Does empathy trigger only altruistic motivation? How about selflessness or justice? Emotion 8, 766–774. 10.1037/a0013967 19102587

[B53] Weir K. Duveen G. (1981). Further development and validation of the prosocial behaviour questionnaire for use by teachers. J. Child Psychol. Psychiatry 22, 357–374. 7287845 10.1111/j.1469-7610.1981.tb00561.x

[B54] Zahn-Waxler C. Radke-Yarrow M. (1982). “The development of altruism: alternative research strategies,” in The Development of Prosocial Behavior, ed. Eisenberg N. (Academic Press), 109–137.

[B55] Zahn-Waxler C. Radke-Yarrow M. Wagner E. Chapman (1992). Development of concern for others. Dev. Psychol. 28, 126–136.

